# Effect of Cold Stress on Neurobehavioral and Physiological Parameters in Rats

**DOI:** 10.3389/fphys.2021.660124

**Published:** 2021-09-17

**Authors:** Hajar El Marzouki, Youssef Aboussaleh, Mohamed Najimi, Fatiha Chigr, Ahmed Ahami

**Affiliations:** ^1^Biology and Health Laboratory, Unit of Clinical and Cognitive-Behavioural Neurosciences and Applied Nutrition Health, Department of Biology, Faculty of Sciences, Ibn Tofail University, Kenitra, Morocco; ^2^Biological Engineering Laboratory, Faculty of Sciences and Techniques, Sultan MoulaySlimane University, Beni Mellal, Morocco

**Keywords:** chronic intermittent cold stress, working memory, anxiety-like behavior, body weight, food intake, sex difference

## Abstract

**Objective:** Cold stress is an important current issue and implementing control strategies to limit its sometimes harmful effects is crucial. Cold is a common stressor that can occur in our work and our occupational or leisure time activities every day. There are substantial studies on the effects of chronic stress on memory and behavior, although, the cognitive changes and anxiety disorders that can occur after exposure to chronic intermittent cold stress are not completely characterized. Therefore, the present study was undertaken with an aim to investigate the effects of chronic intermittent cold stress on body weight, food intake and working memory, and to elucidate cold stress related anxiety disorders using cognitive and behavioral test batteries.

**Methods:** We generated a cold stress model by exposing rats to chronic intermittent cold stress for 5 consecutive days and in order to test for the potential presence of sex differences, a comparable number of male and female rats were tested in the current study. Then, we measured the body weights, food intake and the adrenal glands weight. Working memory and recognition memory were assessed using the Y maze and the Novel Object Recognition (NOR) tasks. While, sex differences in the effects of chronic stress on behavior were evaluated by the elevated plus maze (EPM), open field maze (OF), and Marble burying (MB) tests.

**Results:** We found that 2 h exposure to cold (4°C) resulted in an increase in the relative weight of the adrenal glands in male rats. Given the same chronic stress 5 days of cold exposure (2 h per day), increased weight gain in male rats, while females showed decreased food intake and no change in body weight. Both sexes successfully performed the Y maze and object recognition (OR) tasks, indicating intact spatial working memory performance and object recognition abilities in both male and female rats. In addition, we have shown that stress caused an increase in the level of anxiety in male rats. In contrast, the behavior of the female rats was not affected by cold exposure.

**Conclusion:** Overall, the current results provide preliminary evidence that chronic intermittent cold stress model may not be an efficient stressor to female rats. Females exhibit resilience to cold exposure that causes an increase in the level of anxiety in male rats, which demonstrates that they are affected differently by stress and the gender is an important consideration in experimental design.

## Introduction

Stress is a long-observed physiological reaction that occurs when there is pressure or aggression in our environment and has become popular in recent years. The local servomechanisms as well as the precise and delicate interactions between several systems including the behavioral, neuroendocrine and autonomic responses induced by stressful situations serve to maintain or restore a dynamic equilibrium called homeostasis. However, when the physiological response to stress becomes excessive and prolonged, homeostasis is disturbed and the functioning of body systems is affected, that could trigger adverse health problems related to stress (McEwen, 2000, 2004).

Exposure to a stressful situation affects almost all body systems, especially behavior and physiology. Many studies have demonstrated that stress has a powerful effect on cognitive processes and memory and it may also induce behavioral changes and influence exploratory behavior (Hoffman et al., 2011; Suresh et al., 2013; Santori et al., 2020). Various physiological and behavioral changes following stress have been documented in humans (van der Kolk, 1996) and animals (Bowman et al., 2003).

Changes in exposure temperature may alter the Homeostatic responses. Exposure to extreme temperatures, hot or cold, can produce stress. Cold stress especially has a significant negative impact on the performance and behavior (Solianik et al., 2014) of humans. Extreme Cold exposure affects cognitive function (Lieberman et al., 2009; Shansky and Lipps, 2013) and motor performance (Drinkwater, 2008; Racinais and Oksa, 2010). Moreover, it was reported that even moderate cold stress (0–10°C) may lead to impaired cognitive function (Palinkas, 2001; Mäkinen et al., 2004, 2005, 2006).

Stress can enhance, impair or have no effects on memory. Thus, memory is not considered to be a unitary system and stress can affect it in different ways depending on the memory type tested.

Working memory is considered as the mental structure responsible for temporarily holding and manipulating information and knowledge over brief time periods (Repovs and Baddeley, 2006) and it is also a part of executive functioning necessary for filtering or selecting relevant and inhibiting irrelevant, or no-longer relevant, information (Kane and Engle, 2002), a function that can be especially important in stressful situations. The results concerning the influence of stress on working memory are quite heterogeneous with impaired (Oei et al., 2006; Schoofs et al., 2009), enhanced (Duncko et al., 2009; Cornelisse et al., 2011), or unaffected (Hoffman and al’Absi, 2004; Smeets et al., 2006) working memory capacity.

Exposure to chronic stress may affect memory processes in complex ways depending on the type, duration and intensity of the stress condition or stressor (acute stressors differ from chronic factors), the moment of exposure to stressful stimuli, the specific memory task involved and the age and gender of the subjects (Shors, 2006; Sandi and Pinelo-Nava, 2007; Joëls and Baram, 2009; Bangasser and Shors, 2010).

Most recently, there has been burgeoning interest in the link between stress and anxiety. Chronic stress is thought to be related to a number of mood disturbances (Gold et al., 1988; Sheline, 2000) and might increase the risk of developing depression and anxiety-related disorders (McEwen, 1998; Boscarino and Chang, 1999; Segerstrom and Miller, 2004; Horstmann and Binder, 2011; Popoli et al., 2011; Slavich and Irwin, 2014; Yu, 2016).

Several studies have been performed in order to determine the role of different factors that contribute the most to stress and influence the development of anxious behaviors (Chiba et al., 2012). Among the mental disorders affecting the population, anxiety disorders are the most common and prevalent. Anxiety is associated with disturbances of the internal physiological balance by inducing deleterious effects on the endocrine and nervous systems, and it can also cause biochemical disorders and adversely affect immune responses. Indeed, it has been reported that exposure to stress may induce anxiety disorders (Huynh et al., 2011; Chiba et al., 2012; Solomonow and Tasker, 2015). The biological bases of anxiety disorders depend partly on disturbances in the hypothalamic—pituitary—adrenal axis (HPA axis) (Boyer, 2000). The noradrenergic system has also been implicated in anxiety behaviors (Charney, 2003).

Behavioral changes have been shown to be a significant indicator of stress and have an important role in its assessment. Different tests have been developed and frequently used to assess the effect of stress; standardized behavioral models such as the Open field maze, the Elevated plus maze, and Marble burying test can determine effective changes after exposure to stress including general locomotor activity and exploratory behavior. Although chronic stress produces behavioral changes, animals exposed to acute or chronic stress may exhibit anxiety like-behavior depending on the type and duration of exposure.

Accumulating studies show that males and females exposed to stressors, whether acute or chronic, tend to react and respond to stress differently. Sex differences exist in many aspects, ranging from physiological characteristics such as HPA axis biology, stress response (Kudielka and Kirschbaum, 2005; Bale, 2006; Babb et al., 2013) and chronic stress sensitivity (Kessler and McLeod, 1984; Bebbington, 1996; Hostetler and Ryabinin, 2013); to changes in memory function, learning process and behavior (Weinstock, 2011; Chiba et al., 2012; Suresh et al., 2013; Luine and Gomez, 2015).

In this study, we focus on the sex differences in neurobehavioral and physiological parameters observed in male and female rats following chronic stress. In neurosciences, the majority of animal researches are often conducted on males and females are underrepresented (Beery and Zucker, 2011). Therefore, several findings of the effects of stress can only be provisional until they are confirmed by studies examining the effect of stress on both sexes. However, male and female rats might be affected differently by stress and female rats (show resilient adaptation) are generally more resilient to the effects of chronic stress which can lead to cognitive and behavioral impairment in males.

Although the cognitive and behavioral performance of stressed rodents depends on the type and intensity of stress, cold has been relatively little used as a stressor. Chronic intermittent cold exposure has been applied to induce stress in laboratory animals in order to investigate its effects on different aspects (Dorfman et al., 2009; Lapiz-Bluhm et al., 2009; Girotti et al., 2011; El Marzouki et al., 2014), these studies mainly focused on cognitive and electrophysiological parameters, cold-induced thermogenesis and metabolic responses to cold stress. To our knowledge, there is currently no study reporting the sexually dimorphic effect of intermittent cold stress on working memory and the development of anxiety-like behaviors in rats. Therefore, the aim of the present study was to investigate whether the effects of chronic intermittent cold stress on working memory and behavior are sexually dimorphic, using standardized behavioral models.

## Materials and Methods

### Animals and Stress Exposure Modalities

#### Animal Models

In this study, we used 20 adult male and female rats (3–4 months old) divided into two groups, the control group (*n* = 10, 5 males, 5 females) and the stressed group (*n* = 10, 5 males, 5 females), with five litters per group and one animal per litter in the same experiment group.

Animals were single- housed in Plexiglas cages (30 cm × 15 cm × 12 cm) in a temperature maintained at 22 ± 2°C and a light/dark cycle of 12 h/12 h (8 h00–20 h00). All rats had free access to food and water throughout the experiment.

Experimental procedures on animals were carried out according to approved institutional protocols and in compliance with the guiding principles for the care and use of laboratory animals as described in the Scientific Procedures of Living Animals (European Council directive: ACT: 86/609 EEC).

#### Type of Stress

The stress paradigm used in this study was chronic intermittent cold stress as described previously (El Marzouki et al., 2014). Rats in the cold stressed group were placed in their home cages in a cold room and exposed to 4°C for 2 h, then returned to the housing facility for 5 consecutive days from 8:00 to 10:00 a.m. to avoid corticosterone circadian rhythm. Control rats were kept in their home cages in the housing room and remained undisturbed during this period.

### Physiological Measurements

#### Adrenal Glands Weight

After cold exposure, a series of 5 rats per group were sacrificed by decapitation, the adrenal glands of each animal were removed immediately by laparotomy and weighed in an analytical balance to assess the influence of cold stress on the weight of these organs. The weight of the adrenal gland was used in this study as a parameter of indirect activation of the hypothalamic—pituitary—adrenal axis in response to stress (Rezin et al., 2010).

#### Determination of Plasma Corticosterone Levels

Blood samples were collected from control and stressed male and female rats. Plasma was extracted by centrifugation (3,000 g for 10 min), stored in plastic tubes and frozen until the determination of corticosterone levels. Plasma corticosterone was estimated using a commercial corticosterone ELISA kit.

#### Body Weight and Food Intake

The body weight of each animal and food consumption were measured daily from the beginning of the period of habituation until 1 week after cold exposure.

Every morning, rats were briefly removed from their cages and weighed, and then the residuals were recorded, including the amount of food remaining onto sheets placed under each cage and that which had left on the bottom of the cages. Food intake estimations were calculated by subtracting the weight of food (in grams) recovered from that provided.

#### Behavioral Battery

Behavioral tests were performed on different days to reduce stress which can be induced by the behavioral paradigms themselves and the potential interaction between the tests. Behavioral test battery started the day after the last exposure to cold stress and was performed in the following order: day 1—Open field test; day 2—Elevated plus maze; day 3—Marble burying test; day 4—Y maze; day 5—Novel object recognition test as detailed in the following timeline of the behavioral experiments:

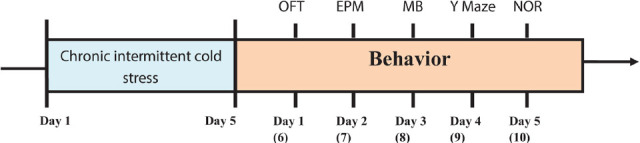



### Cognitive Tests

#### Y-Maze Spontaneous Alternation

The Y Maze Spontaneous Alternation is a behavioral test performed to assess immediate working memory. The protocol of this task is based on the innate tendency characteristic of rodents to explore novelty. The maze used is an apparatus in the form of a capital “Y” consisted of three identical arms (40 × 9 × 16 cm) made of Plexiglas separated from each other by 120° angles. Each rat was placed in the center of the arena and was allowed to freely explore the three arms for 5 min. Normal rats with intact spatial working memory, can remember the arms visited and those not previously explored and show a tendency to explore the least recently visited arm and thus tend to alternate visits between the 3 arms. While a rodent with impairments in working memory failed to remember which arm it has just visited, and thus shows reduced spontaneous alternation behavior (Wall and Messier, 2002). All test sessions were taped through a video camera mounted over the maze. An alternation behavior was operationally defined as successive entries into each of three arms as overlapping triplet sets (i.e., ABC, BCA, CAB etc.). The total number of entries into the arms of the Y maze and the number of triads were recorded in order to calculate the percentage of spontaneous alternation (index of alternation) which is used as an index of spatial working memory performance.

#### Object Recognition Task

The Novel Object Recognition test is a highly validated test used to evaluate cognition in rodents, particularly recognition memory. Before starting the short-term memory task, rats were familiarized by placing them in an empty open field (50 cm × 50 cm × 50 cm) and allowing them to explore it freely. On the next day, rats were placed back into the device and allowed to explore two identical objects (A1 and A2) positioned in two adjacent corners, 10 cm from the arena walls; this was the “acquisition” session. Exploration was considered when rat’s nose touched the object or at least was directed toward it at a distance less than 2 cm. After a delay of 1 h (to evaluate stress effects on short-term recognition memory), each rat was placed in the apparatus and allowed to explore it for 5 min in the presence of the familiar object and a novel object, quite distinct object B. The measurements were taken by using a video camera. Between each trial of recognition testing, the device and the objects were thoroughly cleaned using 70% ethanol to eliminate olfactory cues possibly left by the previous rat (Ennaceur and Meliani, 1988). The amount of time spent exploring the new and the familiar objects provides an index of recognition memory calculated as (time with novel object)/(time with novel object + time with familiar object) ^∗^ 100.

### Behavioral Tests

#### Open Field Test (OF)

The open field test is a behavioral test used to assay anxiety, general activity levels and exploration habits in rodents and it is usually performed in animal experimentation to assess stress and anxiety disorders (Campos et al., 2013). The open field apparatus used was a square arena surrounded by high walls (50 cm × 50 cm × 50 cm) made of Plexiglas, with the floor divided equally into 25 squares (10 cm × 10 cm). At the beginning of the test, each rat was removed from its home cage and placed into the center of the arena and allowed to explore it freely for 10 min. The exploratory behavior of animals was quantified by a videotracking system. The observed behavioral parameters were as follows: the time spent into the central zone (CZ), the time spent into the peripheral zone (PZ), and the number of peripheral and central square crossings. After each test session, the open field box was carefully wiped with ethanol solution (70%) and allowed to dry completely to avoid the presence of any odor traces of the previously evaluated animal.

#### Elevated Plus Maze Test (EPM)

The elevated plus maze test is a well-characterized behavioral paradigm based on rodent’s natural aversion for open areas and can be used to assess anxiety-related behavior in rodent models. As described by Pellow et al. (1985) the apparatus used for this task was a plus (or “X”) shaped maze made of black Plexiglas and consisting of four arms elevated 50 cm above the floor: Two opposite enclosed arms (50 cm × 10 cm × 40 cm) provided with opaque vertical walls and two opposite open arms (50 cm × 10 cm) surrounded by a small a 1-cm high transparent Plexiglas edge which is intended to allow the animal to grip and avoid falling. These four arms were arranged to form a cross whose intersection is called the “central zone” (10 × 10 cm). During behavioral testing, rats were individually placed in the central area with the head facing an enclosed arm and left free to explore the maze for 5 min. The behavior of the rats was recorded using a digital camera placed above the maze. The device was cleaned with ethanol solution (70%) and dried before and after each testing session in order to remove scents throughout the device that may disrupt the performance of the task. The variables identified in the EPM test for the evaluation of anxiety were: the number of entries into the closed and the open arms and the time spent in each of these arms as well as in the central zone.

#### Marble Burying Test (MB)

Marble burying test is an animal model used in scientific research to represent anxiety or obsessive-compulsive disorder behavior. This test consisted of taking rats from their home cage and allowing them to explore another rat cage [45 cm (L) × 23 cm (W) × 20 cm (H)] filled with 5 cm of bedding. Black marbles with a slight metallic sheen were arrayed in a regularly spaced grid in the testing cage (Marks et al., 2009). The cage was kept in a small dark room (1–5 lux). The rats were placed individually into the center of the marble grid and allowed to freely roam in the cage for 20 min without any access to food or water. After the testing period, the rats were returned to their home cages. The latency to bury the first marble and the number of buried marbles were recorded for each rat. Marbles are considered buried if at least 2/3 of the marble is submerged with bedding. Marbles must be washed with soapy water, rinsed with ethanol solution (70%) and then dried after each 20 min trial.

### Statistical Analysis

The Shapiro–Wilk test was used to assess the normality of the data. Non-parametric data (Y-Maze Spontaneous Alternation, Object recognition test, Elevated plus maze, and Open field test) were subsequently analyzed using the Mann–Whitney *U*-test. The variables measured in the Marble burying test were analyzed by the Student *t*-test. The experimental data of body weight and food intake were evaluated using Two-way ANOVA (Treatment × day) followed by Bonferroni’s *post hoc* tests. Statistical treatment was performed using the software program GraphPad Prism (version 5, San Diego, California, United States). The results presented are expressed as the mean ± SEM. and were considered statistically significant when *p*-value < 0.05.

## Results

### Adrenal Glands Weight

**FIGURE 1:**
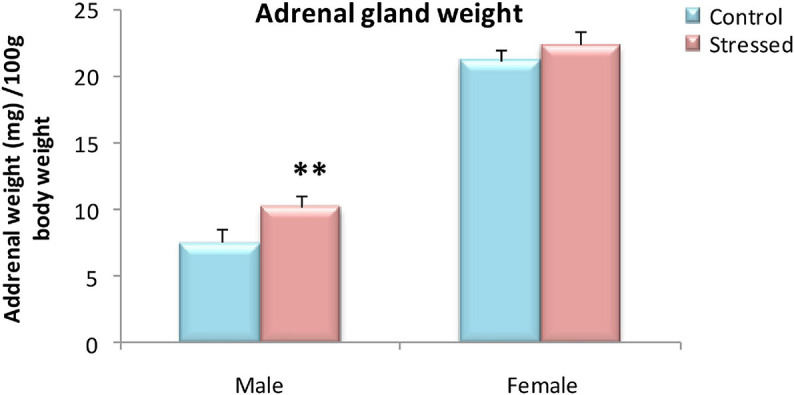
Variation in adrenal weight relative to body weight (mg/100 g of body weight) of control and stressed rats of both sexes. In males, cold stress significantly increased relative adrenal weight in the stressed group compared to the control group. In females, exposure too cold for 5 days had no effect on adrenal glands weight. Adrenal glands weight is expressed in (mg) per 100 g of body weight. Data are represented as mean ± SEM (*n* = 5) for each sex. Effect of stress: ^∗∗^*P* = 0.001.

Daily exposure to 2 h of cold for 5 days resulted in a significant increase in adrenal weight relative to body weight in males, but not in females (Figure 1). The weight of the adrenal glands (mg) was analyzed per 1 g of body weight. In males, analysis with one way ANOVA revealed a significant effect of stress on the adrenal gland/body weight ratio at the time of autopsy (T: 7.54 ± 0.96 mg organ/100 g body weight S: 10.2 ± 0.84 mg organ/g body weight; *P* = 0.001). While, there was no effect of chronic intermittent cold stress on adrenal glands weight in female rats (*P* > 0.05).

### Corticosterone Levels

**FIGURE 2:**
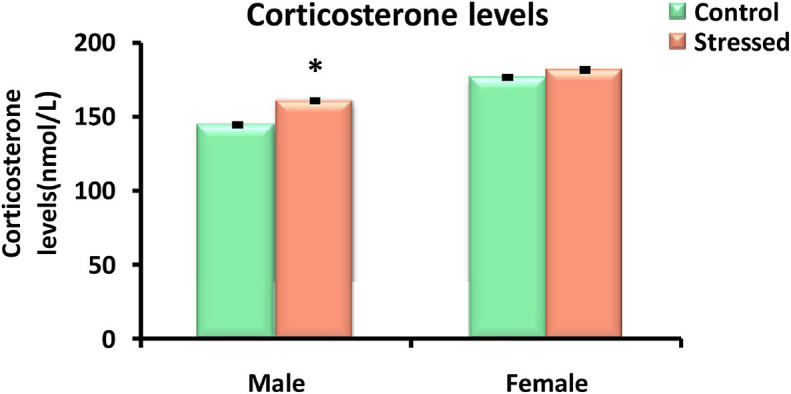
Effect of cold stress on corticosterone levels in male and female rats. Corticosterone was significantly higher in male rats exposed to cold stress compared to their controls. Chronic intermittent cold stress had no significant effect on the corticosterone levels determined in female rats. Data are represented as mean ± SEM (*n* = 5) for each sex. Effect of stress: ^∗^*P* < 0.05.

Plasma corticosterone values were measured in control and cold stressed (4°C, 2 h/day) male and female rats. Exposure to cold stress resulted in a significant elevation in the level of corticosterone in male rats (*P* = 0.012). While, corticosterone levels were not significantly different between stressed female rats and their controls (*P* > 0.05) (Figure 2).

### Body Weight Gain

**FIGURE 3:**
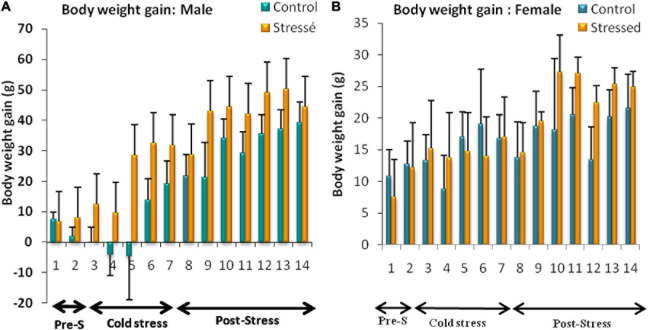
Body weight changes observed in control and stressed rats of both sexes. Cold stress increases body weight gain in male rats **(A)** but does not affect female’s body weight **(B)**. Weight gain is expressed in grams. Data are represented as mean ± SEM (*n* = 5). **(A)** Male rats: Effect of stress: ***p < 0.001; Effect of the day: ****p < 0.0001; Bonfferoni’s *post hoc* test showed no significant interaction between stress and day on body weight gain (*p* = 0.9393). **(B)** Female rats: Effect of stress: ns *p* > 0.05; Effect of the day: ns *p* > 0.05 (ns: not significant).

The results of the weight parameters obtained in our study showed that all male rats continued to grow throughout the experiment, and there was an increase in weight gain in stressed male rats compared to control rats. Two-way ANOVA analysis showed a highly significant effect of stress on body weight gain [*F*_(1,__147)_ = 16.25, *P* < 0.0001], with a highly significant effect of day [*F*_(13,__147)_ = 6.617, *P* < 0.0001].However, Bonfferoni’s *post hoc* test showed no significant interaction between stress and day on body weight gain [*F*_(13, 147)_ = 0.468, *P* = 0.9393] (Figure 3A).

A significant difference between cold-stressed and control female rats was not found. Two-way ANOVA analysis performed on body weight gain in female rats showed no significant stress effect [*F*_(13,__147)_ = 0,734; *P* = 0.393], or day effect [*F*_(1__3,__147)_ = 0.9566; *P* = 0.4969].

The Bonfferoni’s *post hoc* test showed no significant interactive effect of stress and day on body weight gain [*F*_(13,__147)_ = 0.2105; *P* = 0.9985] (Figure 3B).

### Food Intake

**FIGURE 4:**
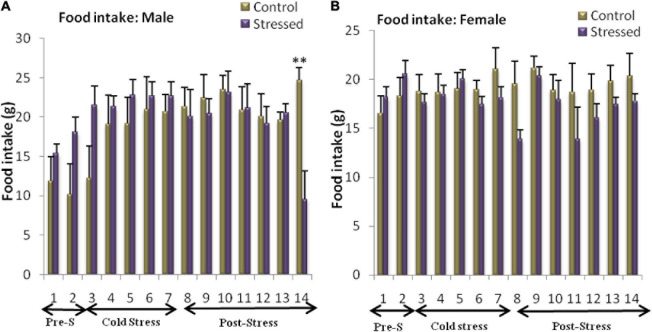
Variation in food intake in control and stressed rats of both sexes. Male rats are not affected by chronic intermittent cold stress until the 14th day (post stress), represented by a decrease in food intake **(A)**. In female rats, cold exposure leads to a significant decrease in food intake **(B)**. Food intake is expressed in grams (g). Data are represented as mean ± SEM (*n* = 5). **(A)** Male rats: Effect of stress: ns *p* > 0.05. Effect of the day: **P < 0.01. (*n* = 5). **(B)** Female rats: Effect of stress: *p < 0.05. Effect of the day: ns *p* > 0.05 (ns: not significant).

Food consumption was measured during and after the period of stress. The main data obtained from food diaries are represented in Figure 4. The analysis carried out on the results obtained in male rats revealed a significant effect of the day on food intake [*F*_(__13,__147)_ = 2.541; *P* = 0.0035], with no significant effect of stress [*F*_(1_, _147)_ = 0.7449; *P* = 0.3895] and a significant interaction between day and stress [*F*_(__13,__147)_ = 2.36; *P* = 0.0068].

Bonfferoni’s *post hoc* analysis showed a significant decrease in food intake in the stressed rat on day 14 (Post stress: *t* = 3.865; *P* < 0.01), this indicates that time affected food intake differently in stressed male rats (Figure 4A).

The two-way ANOVA analysis of the results obtained revealed a significant effect of stress on food intake in female rats [*F*_(__1,__147)_ = 6.432; *P* = 0.0123], with no significant effect of the day [*F*_(3_, _147)_ = 1.216; *P* = 0.2733] or interactive effects of day and stress [*F*_(__13,__147)_ = 1.056; *P* = 0.4022]. The Bonfferoni’s *post hoc* test showed no significant difference between stressed female rats and control female rats on any day of the experiment (Figure 4B).

### Y-Maze Spontaneous Alternation

**FIGURE 5:**
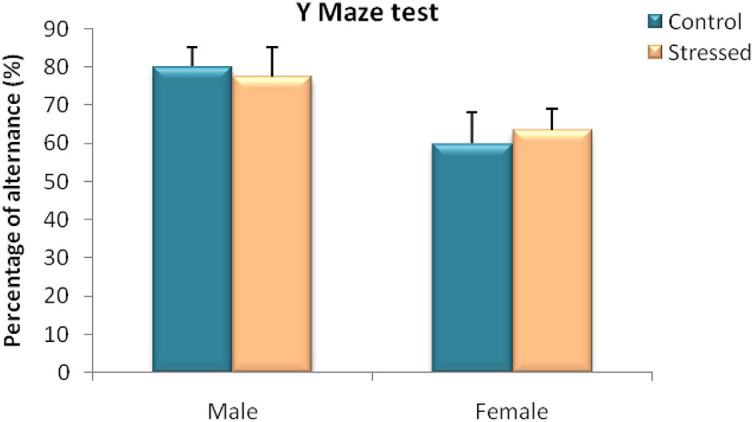
Behavioral evaluation of spatial working memory disorders in male and female rats in a Y-maze spontaneous alternation test (% alternation). In both sexes cold stress had no effect on spatial working memory assessed in Y maze test. Each value represents the mean ± SEM (*n* = 5); Effect of stress: ns *p* > 0.05 (ns: not significant).

In this test, we measured the effect of chronic intermittent cold stress on spontaneous alternation of rats in the Y-maze. Statistical analysis by Mann Whitney test of our results obtained in male rats revealed no significant effect of cold stress on the percentage of alternation of the stressed group in the three arms compared to the control group (*U* = 12; *P* = 1) (Figure 5).

Furthermore, the results of the performance of the female rats during this test did not show any significant difference (*U* = 10; *P* = 0.6905) between the percentage of alternation of stressed female rats (63.57 ± 5.36%) and that of female control rats (59.97 ± 8.07%) (Figure 5).

This indicates that this type of stress had no effect on the immediate spatial memory of male and female rats.

### Object Recognition Test

We used this test to assess the effects of cold stress on the short term object recognition memory in stressed male and female rats and their controls.

**FIGURE 6:**
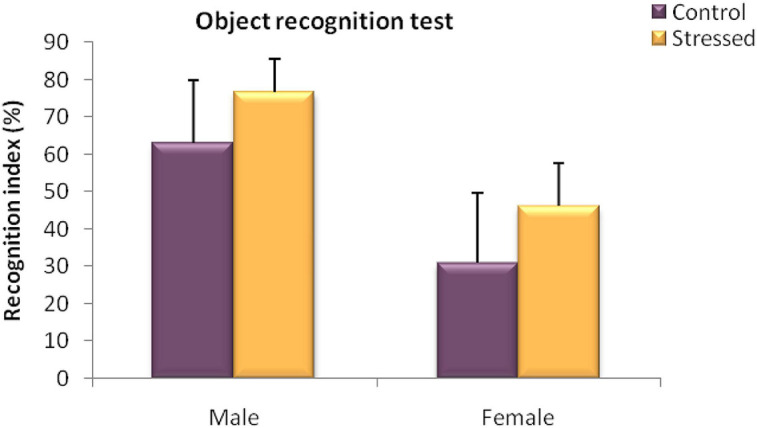
Behavioral evaluation of recognition memory disorders in male rats in a Novel object recognition test (Recognition index%). Cold stress had no significant effect on the recognition performance assessed in an object recognition test in male and female rats. Each value represents the mean ± SEM; Effect of stress: ns *p* > 0.05 (ns: not significant) (*n* = 5).

The results obtained in male rats in this test revealed no significant difference between the recognition index of the stressed group and that of the control group (*U* = 10; *P* = 0.6745) (Figure 6).

Moreover, Statistical analysis of the results obtained in female rats showed no significant effect of exposure to cold stress on the behavior of the animals observed in this test (*U* = 7.5; *P* = 0.344) (Figure 6).

Thus, these findings indicate that chronic intermittent cold stress had no effect on the short term memory assessed with novel object recognition test.

### Elevated Plus Maze Test

**FIGURE 7:**
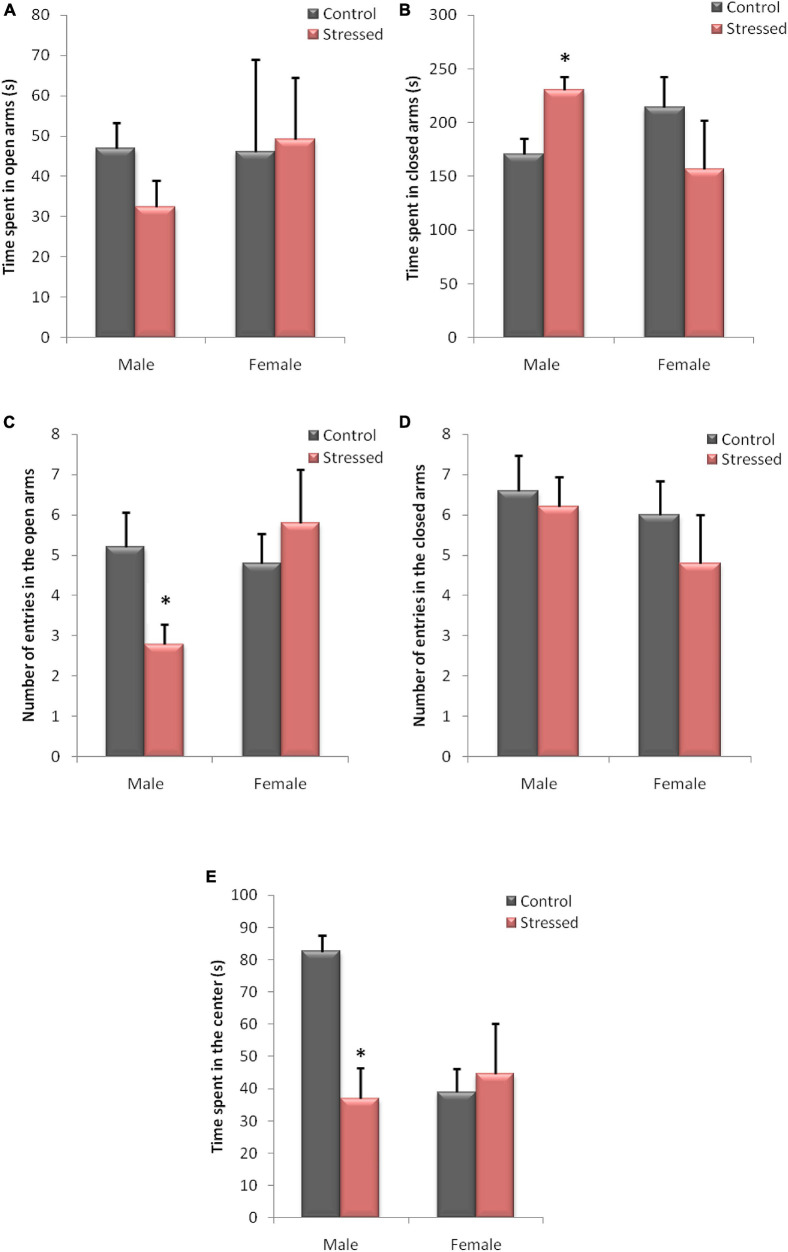
Behavioral evaluation of anxiety-like behavior in male and female rats in an Elevated plus Maze. The behavior of the animals was recorded by a videotracking system. In male rats, chronic intermittent cold stress causes an increase in the time spent in closed arms **(B)**, with a decrease in the time spent in the center **(E)**. It also leads to a decrease in the number of entries in the open arms. Cold exposure had no significant effect on the various variables measured during this test in female rats. **(A)** Time spent in open arms; **(B)** time spent in closed arms; **(C)** number of entries in open arms; **(D)** number of entries in closed arms; **(E)** time spent in the center. Each histogram represents the mean ± SEM; effect of stress: ^∗^ns *p* < 0.05, ns: (not significant) *p* > 0.05 (*n* = 5).

We used this test to evaluate the level of anxiety in male and female rats. Figure 7 shows the main results obtained concerning the EPM test on the time spent (AB) and the number of entries (CD) in the open arms, compared to the closed arms, and also the time spent in the center (E) in male and female rats.

Statistical analysis of the EPM test sessions showed a significant effect of stress in male rats. In this experiment, stressed male rats showed a higher spent time in the closed arms (*P* = 0.0456; *U* = 2.5), and a lower spent time in central area (*P* = 0.0317; *U* = 2) compared to controls. We also observed a reduced percentage of entries into open arms (*P* = 0.0417; *U* = 2.5) following cold stress in this behavioral test. However, the results obtained in EPM did not show a significant effect of cold stress on time spent in open arms (*P* = 0.6905; *U* = 10) and number of entries in closed arms (*P* = 1; *U* = 12).

In contrast, the statistical analysis of the experimental data recorded in female rats in this test showed no significant difference in the time spent in the open arms (*P* = 0.841, *U* = 11), the time spent in the closed arms (*P* = 0.5476, *U* = 9), and the time spent in the center (*P* = 1, *U* = 12). Similarly, differences in the number of entries in open arms (*P* = 0.745, *U* = 10.5), and in the number of entries in closed arms (*P* = 0.5248, *U* = 9) were not observed between stressed female rats and their controls.

### Open Field Test

We used the open-field test to assess anxiety-related behavior in male and female rats.

**FIGURE 8:**
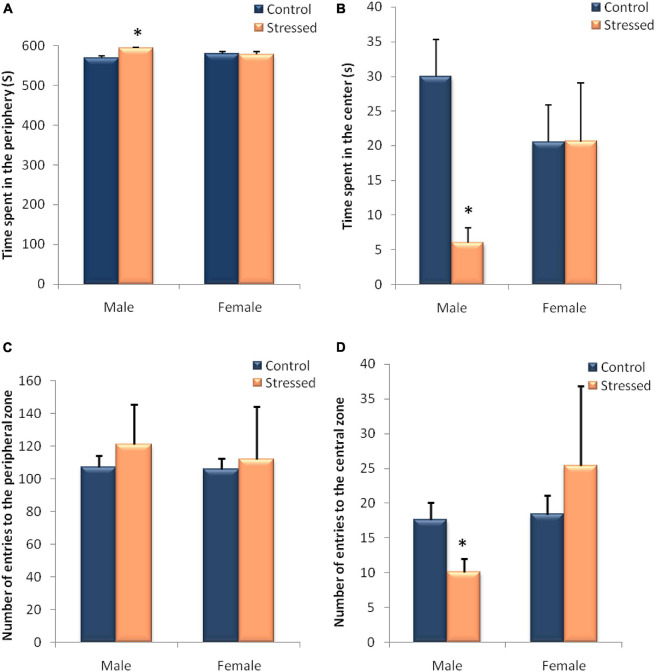
Behavioral evaluation of anxiety-like behavior in male and female rats in an open field test (OF). The behavior of the animals was recorded by a videotracking system. In male rats, exposure to cold leads to an increase in the time spent in the peripheral zone **(A)** associated with a decrease in the time spent in the central zone **(B)** and a decrease in the number of entries in the central zone **(D)**. In female rats, intermittent cold stress had no significant effect on the various variables measured during this test. **(A)** time spent in the peripheral zone; **(B)** time spent in the central zone; **(C)** number of entries to the peripheral zone; **(D)** number of entries to the central zone. Each histogram represents the mean ± SEM. Effect of stress: ^∗^*p* < 0.05 (*n* = 5), ns *p* > 0.05 (ns: not significant) (*n* = 5).

The effects of stress in the open-field behavior in male rats are summarized in Figure 8. The data Analysis of open field test sessions indicated that the time spent in the periphery was significantly higher in the stressed group than in the control group (*P* = 0.0159, *U* = 1), whereas the time spent in the center was significantly lower (*P* = 0.0159, *U* = 1). Furthermore, the number of entries to the central zone was significantly lower in stressed male rats when compared to controls (*P* = 0.0465, *U* = 2.5). Regarding the number of entries to the peripheral zone we noticed no significant differences between stressed and controls male rats (*P* = 1, *U* = 12).

The results obtained in Figure 8 show the behavioral variables recorded during the open field test in stressed female rats compared to control rats. Statistical analysis of these results, showed no significant effect of the cold stress on exploratory behavior recorded in the open field, except that the number of entries to the peripheral zone was higher in stressed female rats when compared to controls (C: 103.8 ± 6.681; S: 111.8 ± 32.53), but this variation did not reach significance (*P* = 1, *U* = 12).

### Marble Burying Test

The final behavioral test was the marble burying which we used to examine if cold stress leads to enhanced anxiety-like behavior in male and female rats.

**FIGURE 9:**
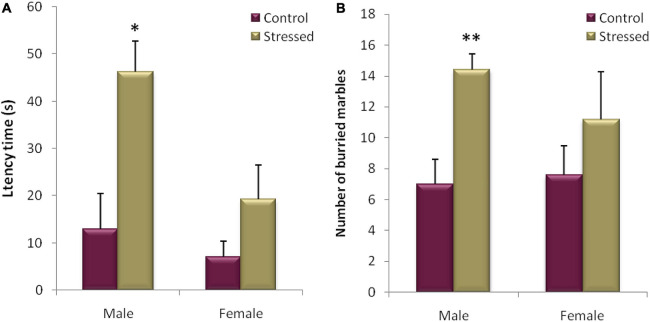
Evaluation of the burying behavior in Marbel Burying test (MB) for 20 min of recording in male and female rats. The cold stress studied leads to an increase in the latency time **(A)**, as well as an increase in the number of buried marbles **(B)** in male rats. In female rats, exposure to cold had no detectable effect on latency time **(A)** and number of buried marbles **(B)** between the two groups of rats. Each histogram represents the mean ± SEM. Effect of stress: ^∗^*p* < 0.05, ^∗∗^*p* < 0.01, ns *p* > 0.05 (*n* = 5).

After the statistical analysis of the results obtained during this test in male rats, a significant difference in the latency time was demonstrated (*P* = 0.010, *t* = −3.332) between the stressed male rats which started to dig the litter after (12.964 ± 7.552 s) and their controls (46.18 ± 6.509 s). In addition, the number of marbles buried by the stressed group was significantly higher compared to the control group (*P* = 0.005, *t* = 3.868) (Figure 9).

While our results did not reveal any significant difference between the stressed female rats and their controls, concerning the latency to initiate burying (S: 19.2 ± 7.276 vs. T: 7 ± 3.406; *P* = 0.167, *t* = −1.519), and the number of marbles buried (S: 11.2 ± 3.056 vs. C: 7.6 ± 1.887; *P* = 0.346, *t* = −1.002) (Figure 9).

## Discussion

The body response to stress is both biological and behavioral. The biological response involves many parameters (cardiovascular, metabolic, immunological, and nervous) and the behavioral response is an adaptation to an unexpected situation allowing the individual to escape as much as possible from the stressor.

Prolonged exposure to stressors may influence feeding behavior and energy homeostasis by inducing various alterations in the amount of food consumed (Epel et al., 2001). Chronic stress can induce morphological variations in animals, and change of body weight might be an important index of physiological responses to stress (Selye, 1976). Body weight change is a part of coping strategies (Uochi and Asashima, 1998; Aoki et al., 2003), which can be an increase (Wang et al., 1995), a decrease (Merritt, 1995), or even no change (Reynolds and Lavigne, 1988) during cold adaptation.

Numerous Studies in laboratory animals under stressful conditions show that food intake is either stimulated or inhibited. The nature of the stimulus, its intensity and its duration determine the observed response. Indeed, stress is known to affect metabolism in a different ways, either by increasing food intake and weight gain (Dallman et al., 2005; Nieuwenhuizen and Rutters, 2008), or by reducing calorie consumption and body weight (Torres and Nowson, 2007; Depke et al., 2008).

Stress-induced alterations in body weight and food consumption are used as a dependable indicator to evaluate the severity of the stress paradigm. Furthermore, stress may have different effects on the human eating behavior; exposure to stressful stimuli resulted in a decrease in food consumption and weight loss in 30% of individuals, while roughly 70% of subjects increased their food consumption (Stone and Brownell, 1994; Epel et al., 2004).

The results obtained in our study showed that chronic intermittent cold stress significantly increased body weight gain in male rats and had no effect on their food intake until the 7th day post-stress. Although these results are in agreement with the data of Chantal and Nieuwenhuizen (Chantal et al., 2003; Nieuwenhuizen and Rutters, 2008) showing a significant body weight gain in stressed rats; they contrast with the earlier reports showing that chronic stress attenuate food consumption and body weight in male rats (Torres and Nowson, 2007; Depke et al., 2008). Cold stress increased body weight gain in male rats without altering their food intake. This result suggests that energy expenditure was reduced by stress. Cold stress seems to potently stimulate NPY secretion (Kuo et al., 2007), that could plays important roles in regulating energy expenditure. It has been demonstrated that increases in NPY activity in the catechominergic system may decrease energy expenditure and induce weight gain during cold stress (Zhang et al., 2014).

Exposure to cold decreased food intake in female rats but did not affect their weight gain for 14 days. These results are consistent with previous studies showing that exposure to stress has minimal or no effect on the body weight of females (Duncko et al., 2001; Trentani et al., 2003; Westenbroek et al., 2003, 2004, 2005; Lin et al., 2008, 2009; Ortiz et al., 2015). However, other studies have found that chronic restraint stress (McLaughlin et al., 2005; Conrad et al., 2012) and chronic unpredictable stress (McFadden et al., 2011) can lead to weight loss. Thus, it seems that female rats are less sensitive to the effects of cold stress on body weight gain than other stress paradigms. Female rats had stabilized body weight despite reduced food intake, suggesting that metabolic adaptations occur during stress, which increases caloric efficiency. The unchanged body weight may be due to the fact that the energy intake is the same as the energy expenditure in which the energy intake is mainly used for thermoregulation.

These changes in body weight and food intake elicited by chronic intermittent cold stress, lead to the conclusion that male and female rats may respond and react differently to this type of stress. Indeed, the stress response can vary depending on many factors such as the type, duration and intensity of stressors and also the sex of laboratory animals (Paré and Redei, 1993; Pucilowski et al., 1993; Martí et al., 1994). Therefore, different stress patterns may have different effects on eating behavior and body weight gain in male and female rats.

The stress response is a set of harmonized, dynamic and complex reactions involving neurochemical, neurobehavioral and physiological processes necessary for the phenomenon of adaptation to a stressful situation. The hypothalamic-pituitary-adrenal (HPA) axis is an important and complex system involved in the regulation of neuroendocrine responses to stress and the adrenal gland which is the organ and the hormonal system most affected during a stress response is part of this axis (Ulrich-Lai et al., 2006).

During exposure to chronic stressors, adrenocorticotropic hormone (ACTH) induces unusual stimulation of the adrenal gland which leads to its hypertrophy (Macedo et al., 2015). Thus, we evaluated the effect of cold exposure on adrenal glands responsivity using their weight as an indirect indicator of HPA axis activation in response to stress, and on corticosterone levels as a potential biomarker of stress in male and female rats.

In the current study, relative adrenal weights and levels of corticosterone were significantly increased in cold stressed male rats compared to their controls. Corroborating our result, Kioukia-Fougia et al. (2002) showed increase in adrenal gland weight in male rats in response to exposure to cold. This stress-related adrenal gland enlargement was not a surprising discovery, as the adrenals have been shown to have one of the highest rates of blood supplies per gram of tissue during exposure to stress (Hornsby, 1985). Thus, adrenal hypertrophy and higher levels of corticosterone found in male rats can be interpreted as a result of reaction to a stressful situation, indicating the activation of the HPA axis, known as a physiological system extremely sensitive to conditions of stress (Kenjale et al., 2007), which causes the secretion of adrenocorticotrophic hormone (ACTH) and corticosterone from the adrenal glands to help the body cope with stress (Zafir and Banu, 2009).

In female rats, relative adrenal glands weights and corticosterone levels were unaltered following chronic intermittent cold stress, suggesting a potential adaptation of the hypothalamic-pituitary-adrenal axis after repeated exposure to the same stressor (Viau and Sawchenko, 2002).

Overall, these results showed that chronic intermittent cold stress affects physiological parameters assessed in male rats in a similar way to that of other types of chronic stress. Unlike males, female rats exposed to cold did not exhibit body weight change, hypertrophy of the adrenal gland or elevated levels of corticosterone. Therefore, the effectiveness of cold stress in male rats was affirmed by body weight gain, enlarged adrenal gland, and higher levels of plasma corticosterone. While female rats showed resistance to the evaluated physiological impacts of cold stress although they exhibited decreased food intake.

Stress can contribute to behavioral modifications and it is thought to be responsible for many neuropsychiatric disturbances such as depression or anxiety disorders on human (Garcia-Bueno et al., 2008). It has been shown that exposure to stressful conditions induces anxious and depressive behavior in rodents (Haenisch et al., 2009; Hageman et al., 2009; Regenthal et al., 2009; Huynh et al., 2011; Chiba et al., 2012). However, stress does not always lead to behavioral changes (Gregus et al., 2005; Swiergiel et al., 2007). There have been several studies on the effects of various types of stressors on behavior in male rats. In contrast, to our knowledge, there has been little research on the effects of cold stress on the behavior of male and female rats.

Cold stress may have its own specific pattern of neurobehavioral alterations. Thus, we evaluated the effects of cold environment on the neurobehavioral functions of rats by proposing different behavioral tests to assess the level of anxiety among male and female rats.

Cold Stressed male rats demonstrated enhanced anxiety as shown by an increase in time spent in the outer zone and a reduction in time spent in the inner zone of the open field, suggesting decreased motivation to explore a new environment due to higher levels of anxiety in stressed rats (Tobach, 1966; Katz et al., 1987). These findings are consistent with the studies undertaken by Veenema and Neumann (2007); Chiba et al. (2012), and Suresh et al. (2013) which report that stress can cause aggressiveness, and it induces in particular an increase in the level of anxiety assessed in the open-field test.

Whereas female rats exposed to cold did not show stress-induced anxiety behavior changes, this indicates that the stress applied in our study did not affect the level of anxiety assessed in a new environment in female rats. A result that is not supported by previous studies showing that chronic stress has been found to increase anxiety-like behavior assessed in the open field test (OF) (Bowman and Kelly, 2011; Manikandan et al., 2015).

To further confirm the development of anxiety-related behavior in rats in response to cold stress, we also used the elevated plus maze test (EPM), which is a validated to assess emotional response particularly anxiety (Pellow et al., 1985; Rodgers and Cole, 1993a; Ohl et al., 2001; Walf and Frye, 2007). The behavior of animals in the elevated plus maze can be influenced by stress factors such as cold (Hata et al., 2001) electric shocks (Steenbergen et al., 1990) and forced swimming (Britton et al., 1992).

Our data, demonstrated that stressed male rats spent a greater amount of time exploring the closed arms than the open arms of the elevated plus maze and made fewer entries into the open arms, reflecting higher levels of anxiety associated with exposure to cold. These results are in agreement with previous studies in males showing that chronic stress induces anxious and depressive behavior in rodents (Huynh et al., 2011; Chiba et al., 2012; Suresh et al., 2013; Manikandan et al., 2015), and it also generates behavioral changes in humans and can lead to anxiety disorders (Garcia-Bueno et al., 2008; Horstmann and Binder, 2011).

Chronic intermittent cold stress, on the other hand, did not affect the parameters measured on the elevated plus maze task in female rats. Although this finding is consistent with other studies showing that stress has no effect on the level of anxiety assessed in (EPM) in female rats (Rodgers and Cole, 1993b; Marcondes et al., 2001; Voikar et al., 2001; Bowman et al., 2009); resistance to chronic stress observed in female rats disagrees with the anxiogenic effect reported by Beck and Luine (2002); Bowman et al. (2004), Gregus et al. (2005); Swiergiel et al. (2007), Huynh et al. (2011), and Manikandan et al. (2015). Contradictory findings on behavioral responses to stress in female rats evaluated with the OF and EPM tests are probably due to the difference in stressors applied, the duration of the stress and the experimental procedures (e.g., day—night; the method of applying the stress) and genetic factors (Huynh et al., 2011).

Marble burying is another rodent model employed to study anxiety disorders (Jury et al., 2015; Silverman et al., 2015; Ene et al., 2016). The elevated plus maze (EPM) and the open field (OF) models are based on the test animal’s aversion to open and brightly lit areas, while the Marble burying test is applied to evaluate fear of a new aversive materiel. Rodents have an inherent tendency to bury either harmful (Koolhaas et al., 1999; Pinel et al., 1994) or non-harmful (Njung’e and Handley, 1991; De Boer and Koolhaas, 2003) objects in their bedding, to protect against the potential danger posed by the object.

Behavioral assessment of anxiety-like behavior in a Marble burying test revealed that female rats exposed to chronic intermittent cold stress for 2 h per day showed no difference in the burying behavior compared to control female rats. Whereas male rats that received the same type of stress hid more number of marbles than control male rats, reflecting an increase marble-burying behavior. Indeed, the number of hidden marbles is directly related to the response to anxiety, indicating that cold stress increased the level of anxiety in male rats while it had no effect on anxiety-related behavior in female rats.

Our results obtained in this series of behavioral experiments showed that unlike males, female rats do not exhibit anxious behavior in new environments. Although it was reported that anxiety is more upon in stressed female animals (Bowman and Kelly, 2011; Huynh et al., 2011; Manikandan et al., 2015), this was not the case in cold stressed female rats. This result has been interpreted as reflecting an increased capacity to cope with stressful situations known as resilience and, thus, a decreased vulnerability to behavioral disorders or alterations induced by cold stressors.

This difference between male and female rats is probably attributable to the fact that male rats are vulnerable in HPA axis deregulation after exposure to chronic cold stress. The cumulative load of repeated cold stress resulting in high level of anxiety, thus, the male rats could not adapt and get used to repeated stress and could not display an anxiolytic state. While female rats might adapt to repeated cold stress and show no signs of anxiety in a new environment. These resulting data provide new information on the effects of cold stress on levels of anxiety-related behaviors in female rats.

It has been proven that males and females rats respond differently to stress, and this sexual dimorphism in the behavioral response might be related to the effect of sex hormones and gonads effect on the brain etc. (Renard et al., 2005). Our study demonstrates that anxiety-related behaviors differ between males and females after exposure to stress, thus, cold stress response is also sexually dimorphic.

The activation of the HPA axis by acute stress is considered an adaptive biological response intended to cope with stressful situation. While, chronic stress may lead to dysregulation of the hypothalamic-pituitary-adrenal axis resulting in an increased risk of disease or health disorders (de Kloet et al., 2005). In fact, a relationship appears to exist between the HPA axis hyperactivity and the development of anxiety disorders (Martin et al., 2010; Bangasser and Valentino, 2014).

Our data indicate that, male rats were more vulnerable in the application of chronic cold stress than female rats. These differences obtained in stressed rats are probably due to the sex-related differences in stress-induced activation of the HPA axis (Patchev and Almeida, 1998; Young, 1998) and its reciprocal relationship with serotonergic function (Heninger, 1997; Joffe and Cohen, 1998). Behavioral differences between male and female rats have been linked to HPA axis functioning and serotonergic system which may respond differently in the two sexes after acute (Karandrea et al., 2002; Drossopoulou et al., 2004) or chronic (Duncko et al., 2001; Beck and Luine, 2002; Bowman et al., 2003, 2009; Konkle et al., 2003; Westenbroek et al., 2003) stressful conditions.

Exposure to cold stress triggers a reduction in the serotonin levels in most regions of the brain in male rats (Toh, 1960; Aly et al., 1985; Hata et al., 1991) and the serotonergic system is thought to be implicated in the modulation of anxiety (Graeff et al., 1996; Voigt et al., 1999; Holsboer, 2000; Millan, 2003). In this regard, it has been reported that reduced stress-induced hippocampal 5-HT release is observed in rats that display high levels of anxiety (Keck et al., 2005). In fact, reduction in ventral hippocampal 5-HT levels is believed to increase anxiety-like behavior (Tu et al., 2014) and enhancement of 5-HT concentrations in the hippocampus contributes to reduce it (Guimaraes et al., 1993; Graeff et al., 1996), indirectly suggesting that hippocampal serotonin levels are involved in adaptive coping. The anxiety-like behavior observed in cold-stressed male rats may thus be related to the disruption of the serotonergic system.

In addition, a persistent increase in corticosterone (CORT) levels may be an important factor that can increase the risk of developing an anxiety disorder in stressed male rats. This interpretation is supported by higher levels of corticosterone and increased adrenal weight observed in these rats, which indicates hyperfunction of the adrenals due to chronic stress (Dallman et al., 1992; Blanchard et al., 1998).

Elevated CORT levels can increase anxiety-like behavior in rats by several mechanisms, among which we suggest the possibility of serotonin dysregulation. This hypothesis is supported by studies demonstrating that chronic administration of exogenous corticosterone lead to alterations in serotonergic function and expression of 5-HT1A and 5-HT2A receptors in male rats (Dickinson et al., 1985; Fernandes et al., 1997; Gorzalka and Hanson, 1998; Karten et al., 1999).

On the other hand, sex differences in anxiety-like behavior observed in the present study can be explained in part by the influence of gonadal hormones. Stress appears to be a potential risk factor for reproductive function, one of the known consequences of stress is the decline in male fertility (Clarke et al., 1999). The activation of the HPA axis in response to stress can inhibit the normal functioning of the male reproductive system via suppression of the hypothalamic-pituitary-gonadal axis (HPG axis) (Ferin, 2006). Exposure to stressful conditions has been shown to limit sperm production, impair spermatogenesis, reduce sperm counts and motility, increase amounts of morphologically abnormal sperm and decrease testosterone and LH levels (Almeida et al., 2000; Khandve et al., 2013). Thus, higher levels of anxiety in male rats may also be a reflection of stress-induced inhibition of testosterone secretion. Testosterone replacement or administration induces anxiolytic-like effects in castrated male rats (Fernandez-Guasti and Martinez-Mota, 2005) and increases the number of entries in the open arms in elevated plus maze test (EPM) (Bitran et al., 1993; Frye and Seliga, 2001), suggesting that anxiety-like behavior is associated with lower testosterone levels.

The different response in female rats is probably due to their hormonal adaptation to cold stress (Suresh et al., 2013; Wang et al., 2015) as well as the differential action of sex steroids (estrogen and progesterone) and their organizational and activational effects on behavioral responses to stress (Patchev and Almeida, 1998; Rachman et al., 1998; Karandrea et al., 2002; Bowman et al., 2003; Dalla et al., 2004).

Indeed, stress-induced changes including anxiety related behavior might be influenced by the reproductive cycle of female rats (ter Horst et al., 2012). During proestrus (estradiol and progesterone are high) and estrus (peak estrogen secretion) phases, female rats spend more time in the open-arms of the elevated plus maze (EPM) than rats in diestrus (low estradiol), indicating decreased anxiety-like behavior (Mora et al., 1996; Díaz-Véliz et al., 1997; Frye et al., 2000; Marcondes et al., 2001; Gouveia et al., 2004; Walf et al., 2009).

Furthermore, researches in animal models suggests important roles for progesterone and the neuroactive steroid allopregnanolone in stress and anxiety. Thus, the decreased anxiety level in female rats might be related to the regulatory effects of progesterone and allopregnanolone. In females, high levels of circulating progesterone are converted to allopregnanolone. Therefore, higher levels of allopregnanolone under stressful conditions lead to lower levels of anxiety in female rats than in male rats (Zimmerberg and Brown, 1998; Kelly et al., 1999). In addition, the open arms exploration in elevated plus maze is increased by allopregnanolone administration to female rats, indicating a decreased anxiety levels (Zimmerberg and Brown, 1998).

Working memory is a cognitive system responsible for the storage and manipulation of information for a brief period of time (Repovs and Baddeley, 2006). Stress affect memory processes in complex ways, and its effect depends on many factors such as the type and the duration of stress, the moment of applying the stressor and the cognitive tasks used (Joëls and Baram, 2009).

In the present study, spontaneous alternation behavior, which is considered to reflect spatial working memory, was assessed in the Y-maze by allowing rats to freely explore the three arms of the maze and this behavior is motivated by the rodents’ innate curiosity to explore previously unvisited areas (Lalonde, 2002). Cognitive profiling of the male and female cold stressed rats revealed that chronic intermittent cold stress had no effect on performance in the spontaneous alternation test, indicating intact working memory ability.

Although scant, there are some previous studies in male rats showing that chronic stress does not alter spatial working memory, but the present data greatly extend these findings. Yet, it should be underscored that while our current findings are in agreement with some previous studies showing that chronic stress had no effect on working memory (Luine et al., 1994, 1996; Williams et al., 1998) or may be causing impaired memory retrieval while leaving working memory intact (Srikumar et al., 2006, 2007), they are inconsistent with previous findings reported by Mizoguchi et al. (2000) and Manikandan et al. (2006) showing that chronic stress impaired spatial working memory or both reference and working memory. This could be due to methodological differences, such as the type and duration of stress and the memory test used.

On the other hand, spatial working memory disorders might be related to the post-stress delay to evaluate the performance of rats in the task. In chronic stress exposed male rats (restraint stress, 6 h per day, for 7 days) tested in the radial arm maze (RAM) with various delay conditions, 10–13 days after stress exposure (Luine et al., 1996) or from 21 days post-stress (Hoffman et al., 2011), no effect on spatial memory performance was observed. While working memory assessed immediately after exposure to stress in the same task (RAM) in male rats was impaired (Hoffman et al., 2011).

The findings of stress related-changes in learning and memory in rats are well documented, but the effect of chronic stress on spatial working memory in females is still in its infancy and further studies will be necessary to investigate stress effect on female spatial working memory.

To our Knowledge, no study on the effects of cold stress on working memory in female rats has as yet been conducted. As the first study, we demonstrated that spatial working memory appear to be not altered in female rats exposed to cold. While, our previous findings (El Marzouki et al., 2014) indicate that chronic intermittent cold stress procedure as administered in our laboratory lead to enhanced spatial learning in female rats and impaired memory retrieval in male rats.

Sex differences have been reported in non-spatial memory in rats exposed to stress. In general, memory performance is not affected by chronic stress in a variety of non-spatial tasks in female rats, but it is impaired in males. In this study, chronic stress does not alter short-term object recognition memory assessed using the object recognition test in male and female rats, suggesting intact recognition memory following cold exposure.

Thus, the findings from the current study are in agreement with several other studies (Beck and Luine, 2002; Bisagno et al., 2004; Bowman et al., 2009; Bowman and Kelly, 2012; Gomez and Luine, 2014) showing that chronic stress has no effect on female performance on the task of object recognition. Although, our surprising results in male rats are inconsistent with previous research (Beck and Luine, 2002; Bowman et al., 2009; Gomez et al., 2013; Santori et al., 2020) indicate that stressed male rats are unable to make a significant distinction between known and new objects following chronic stress exposure, and show impaired object recognition memory; the effect of stress on recognition memory observed in male rats is consistent with these reported by Beck and Luine (1999) suggesting that chronic restraint stress only impairs object recognition memory when the retention interval exceeds 1 h.

Given the fact that recognition memory performance was not altered at shorter delays, cold stress may not have changed the extraneous factors that could influence recognition memory (such as tendency for exploration, locomotor activity, or preferences for novelty) (Baker and Kim, 2002).

Moreover, chronic stress has been associated with impaired memory functions in male rodents. However, these effects may differ depending on the type and intensity of stress, the type of the memory task involved (Conrad, 2010) and the short-delay or long-delay memory tasks (Beck and Luine, 1999).

Indeed, anxiety is most often assessed after exposure to chronic stress, and increased anxiety-like behavior can further disrupt memory function, particularly in exploration-based tests like recognition memory task (Luine et al., 2017). Thus, since cold stress did not alter the cognitive function in both sexes but had a different effect on anxiety; male rats exhibited elevated anxiety-like behavior, while the behavior of the female rats was not affected, anxiety-dependent changes do not appear critical in the cognitive performance.

As with the majority of studies, there are two limitations in the current study that could be addressed in future research. The first is the measurement of Adrenocorticotropic hormone and plasma catecholamine concentrations. The second limitation concerns the evaluation of the appetite related hormones levels such as ghrelin and leptin.

## Conclusion

Cold stress is a collection of physiological and neurobehavioral changes, resulting from repeated exposure to extreme cold conditions and may lead to impaired cognitive functions and behavioral disorders. This study showed that chronic intermittent cold stress causes an increase in body weight and relative adrenal gland weight only in male rats but not in females suggesting potential adaptation of the HPA axis. Cold stress does not have any impact on the performance of spatial working memory and object recognition memory in male and female rats. In addition, exposure too cold for 2 h per day is sufficient to induce anxiety-like behavior in male rats, but it does not affect female rat’s behavior.

We can therefore conclude that female rats show resilience to chronic intermittent cold stress that impairs male behavior, suggesting that they are affected differently by this type of stress. Although the mechanism behind the vulnerability of male rats and the resilience of female rats to chronic intermittent cold stress remains to be studied, this study provides additional evidence on how chronic intermittent cold stress affects the physiology of the organism, and important information about sex differences in cold stress response which emphasizes the influence of gender in experimental design. Thus, future studies are aimed at further evaluating the influence of steroid hormones on cognition and behavior following exposure to cold stress with concurrent elucidation of biochemical mechanisms.

## Data Availability Statement

The raw data supporting the conclusions of this article will be made available by the authors, without undue reservation.

## Ethics Statement

The animal study was reviewed and approved by the Scientific Procedures of Living Animals (European Council directive: ACT: 86/609 EEC).

## Author Contributions

HE conceived and designed the study, performed all the experiments, extracted and analyzed the data, interpreted the results, and wrote the manuscript. YA conceived and designed the study, revised, and approved the final manuscript. MN and FC conceived, designed the study, and supervised the experiments. AA revised the manuscript and provided good advices. All authors contributed to the article and approved the submitted version.

## Conflict of Interest

The authors declare that the research was conducted in the absence of any commercial or financial relationships that could be construed as a potential conflict of interest.

## Publisher’s Note

All claims expressed in this article are solely those of the authors and do not necessarily represent those of their affiliated organizations, or those of the publisher, the editors and the reviewers. Any product that may be evaluated in this article, or claim that may be made by its manufacturer, is not guaranteed or endorsed by the publisher.
